# Targeting Insect Olfaction *in vivo* and *in vitro* Using Functional Imaging

**DOI:** 10.3389/fncel.2022.839811

**Published:** 2022-02-24

**Authors:** Fabio Miazzi, Kalpana Jain, Sabine Kaltofen, Jan E. Bello, Bill S. Hansson, Dieter Wicher

**Affiliations:** ^1^Department of Evolutionary Neuroethology, Max Planck Institute for Chemical Ecology, Jena, Germany; ^2^Department of Entomology, University of California, Riverside, Riverside, CA, United States

**Keywords:** insect olfaction, *Drosophila melanogaster*, olfactory sensory neuron, calcium imaging, silica hydrogel, waterglass

## Abstract

Insects decode volatile chemical signals from its surrounding environment with the help of its olfactory system, in a fast and reliable manner for its survival. In order to accomplish this task, odorant receptors (ORs) expressed in olfactory sensory neurons (OSNs) in the fly’s antenna process such odor information. In order to study such a sophisticated process, we require access to the sensory neurons to perform functional imaging. In this article, we present different preparations to monitor odor information processing in *Drosophila melanogaster* OSNs using functional imaging of their Ca^2+^ dynamics. First, we established an *in vivo* preparation to image specific OSN population expressing the fluorescent Ca^2+^ reporter GCaMP3 during OR activation with airborne odors. Next, we developed a method to extract and to embed OSNs in a silica hydrogel with OR activation by dissolved odors. The odor response dynamics under these different conditions was qualitatively similar which indicates that the reduction of complexity did not affect the concentration dependence of odor responses at OSN level.

## Introduction

In order to build their representation of the external world, animals must acquire and integrate a plethora of different visual, auditory, tactile, magnetic and chemical stimuli. Chemoreception, i.e., the detection of volatile and non-volatile compounds by olfaction and taste, respectively, plays a pivotal role in the ecology for example of nematodes, vertebrates and insects. With the use of genetic tools such as the GAL4-UAS system, it became possible to study molecular mechanisms in insect physiology (Brand and Perrimon, 1993). Especially in the vinegar fly *Drosophila melanogaster* the GAL4-UAS system is extensively used to study tissue specific gene expression and function due to advantages such as short life cycle and complete availability of genomic sequence (Jennings, 2011). For *Drosophila*, olfaction – as for other insects – is the main sense to assess food, mates, oviposition sites and to avoid perils (Hansson and Stensmyr, 2011; Mansourian and Stensmyr, 2015). Volatile compounds in particular are detected by the two main olfactory organs of the fly: the antennae and the maxillary palps. They are covered by porous hair-like structures called sensilla, each one housing the dendrites of up to four olfactory sensory neurons (OSNs). They possess three main classes of olfactory receptors: the odorant receptors (ORs), the ionotropic receptors (IRs), related to the ionotropic glutamate receptors and specific gustatory receptors (GRs), which in the antennae detect, e.g., carbon dioxide (Touhara and Vosshall, 2009; Joseph and Carlson, 2015). ORs are used to perceive a variety of compounds, from food odors and harmful bacterial contaminations to pheromones (Mansourian and Stensmyr, 2015). These receptors are heteromers composed of a ubiquitous odorant co-receptors (Orco) and a neuron-specific receptor (OrX) (Larsson et al., 2004; Neuhaus et al., 2005; Benton et al., 2006). They share a seven transmembrane-domain topology, but unlike canonic G protein-coupled receptors they show an inverted topology with an intracellular N-terminus (Benton et al., 2006; Lundin et al., 2007; Smart et al., 2008; Tsitoura et al., 2010). ORs form ligand-gated cationic channels (Sato et al., 2008; Wicher et al., 2008), which are regulated by multiple metabotropic cascades influencing for example the receptor sensitivity (Nakagawa and Vosshall, 2009; Wicher, 2015).

*In vivo* studies on insect olfaction have greatly benefited from the position of these organs, which are extrovert and easily accessible in the fly’s head, in comparison to the mammalian olfactory epithelium, which is deeply buried inside the nasal cavity. This allowed researchers to perform comprehensive odor-response profile screenings from antennal and palp sensilla using extracellular electrophysiological recordings (de Bruyne et al., 2001; Dobritsa et al., 2003; Hallem et al., 2004; Dweck et al., 2016). On the other hand, imaging experiments from these organs suffer from a limited space resolution, particularly using epifluorescence microscopy, due to the light scattering induced by the cuticle and the sensory hairs. Reports on cellular, functional imaging and patch clamp from OSNs are based on antennal preparations and involved in slicing the antenna and fixing it on a support via silicon-based media and delivering stimuli in water solutions (Mukunda et al., 2014, 2016; Cao et al., 2016). Such approaches are certainly limited due to the invasive techniques used to prepare the samples, but their success clearly shows their potential and urges for further improvements to refine them to and expand the range of biological questions that is possible to address. Insect ORs are also heterologously expressed in *in vitro* and *in vivo* systems to study functional properties and identify specific ligands (Fleischer et al., 2018). For example, the *in vivo* expression system “*Drosophila* empty neuron” allows the ectopic expression of ORs in *Drosophila* OSNs by removing the native OrX protein (Dobritsa et al., 2003; Kurtovic et al., 2007; Gonzalez et al., 2016). Insect OR function is also studied in *in vitro* expression systems as Human Embryonic Kidney (HEK) 293 cells (Große-Wilde et al., 2007; Sato et al., 2008; Wicher et al., 2008; Corcoran et al., 2014). Mammalian HeLa cells (Sato et al., 2008), *Spodoptera frugiperda* Sf9 cells (Kiely et al., 2007), and also *Xenopus laevis* oocytes (Wagner et al., 2000). However, such systems also bear the risk of a low expression level, inadequate protein translation or impaired OR trafficking to the plasma membrane.

In this study, we present preparations of different complexity to study the function of *D. melanogaster* OSNs. We first present an *in vivo* preparation that allows the OSN stimulation with airborne odors, i.e., to study odor-induced responses elicited under natural conditions. We then developed a technique to isolate vital *Drosophila* OSNs from antennal tissue. To allow further functional studies on these isolated cells we established a new embedding method based on sodium silicate (also called waterglass) (Avnir et al., 2006; Pierre and Rigacci, 2011) without any need of greasy or silicon media, which may affect the odor delivery and the response temporal properties. Finally, we offer proof of principle of the effectiveness of such preparations and we discuss their potential to advance our knowledge about olfactory transduction mechanisms in insects.

## Materials and Methods

### Insect Rearing

*Drosophila melanogaster* with genotype (*w, UAS-GCaMP3.0; +; Or22a-Gal4*), (*w; UAS-GCaMP6f; Orco-Gal4*), (*w; UAS-GCaMP6f; Or22a-Gal4*), and (*+; Orco-Gal4/CyO; UAS-Syn21-GFP-p10/TM6B*) were reared on conventional cornmeal agar medium under a 12 h light: 12 h dark cycle at 25°C. UAS-GCamP3.0; Orco-Gal4; UAS-GCaMP6f parental line was obtained from Bloomington Stock Center (#32234), (#42747), (#26818), and Or22a-Gal4 line was kindly provided by Dr. Leslie Vosshall, The Rockefeller University.

### Chemicals

VUAA1 (N-(4-ethylphenyl)-2-((4-ethyl-5-(3-pyridinyl)-4H-1,2,4-triazol-3-yl)thio)acetamide) was synthesized by the group “Mass Spectrometry/Proteomics” of the Max Planck Institute for Chemical Ecology (Jena, Germany). Ethyl hexanoate (99%, Sigma-Aldrich), L-Cysteine hydrochloride (Cat. Nr. C1276, Sigma-Aldrich), Papain (Cat. Nr. 5125, Calbiochem, San Diego, CA, United States), cOmplete protease inhibitor cocktail (Cat. Nr. 04693116001, Roche, Basel, Switzerland), methanol (≥99.5%, Roth) HCl (≥32%, Sigma-Aldrich), H_2_SO_4_ (95∼97%, Sigma-Aldrich), sodium metasilicate solution (Cat. Nr. 13729, Sigma-Aldrich), low Ca^2+^-Schneider’s medium (Cat. Nr. S9895, Sigma-Aldrich), Benzaldehyde (Acros Organics, NJ, United States) were used in following experiments.

### Functional Calcium Imaging From an *in vivo* Antennal Preparation

**FIGURE 1:**
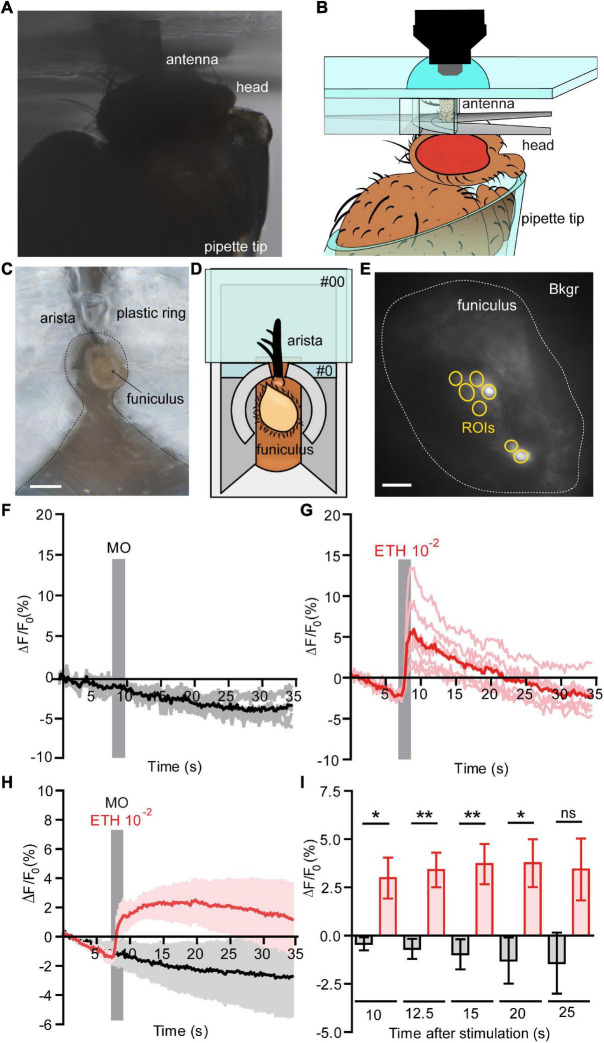
Functional calcium imaging from an *in vivo* antennal preparation. **(A)** Image of the *in vivo* preparation from the side view. **(B)** Schematic representation of the fly placed in a pipette tip with it’s excised antenna held in vertical position on a custom holder, for the detailed information see Supplementary Figure 1. **(C)** Image of the *in vivo* preparation from a top view, Scale bar = 0.5 mm. **(D)** Representation of the *in vivo* preparation from a top view, where open antenna is held within a #0 glass coverslip with support of an aluminum foil holder and a plastic ring around the antenna. A posterior slit on the plastic ring allowed fixing the arista directly on the #0 coverslip with odor-free glue. **(E)** Normalized fluorescence base level intensity from a fly preparation expressing GCaMP3.0 in Or22a olfactory neurons. The regions of interest (ROIs) are marked in yellow and the area used for the background subtraction (Bkgr) are marked in white, Scale bar = 10 μm. **(F,G)** Example of the recorded fluorescence intensity (expressed in ΔF/F0) over time from the same preparation as shown in **(D)**. **(F)** The fly was first stimulated with mineral oil (MO, negative control); the stimulus duration is marked with a vertical gray bar, each ROI as in **(E)** is represented in gray and the mean value in black. **(G)** The fly was then stimulated with ethyl hexanoate (EH) at a 10^–2^ dilution in mineral oil; the stimulus duration is marked with a vertical gray bar, each ROI as in **(E)** is represented in light red and the mean value in red. **(H)** Pooled responses from *n* = 5 antennae to MO (black) and EH 10^–2^ (red). Traces represents mean ± SEM. **(I)** Intensity of the responses to MO (black) and EH 10^–2^ (red) calculated subtracting the fluorescence value at the moment of stimulation (7 s) from the fluorescence intensity at a given time expressed in seconds after stimulation. The response to EH is long lasting and is statistically significant until 20 s after stimulation [correspondent to time = 27 s plotted in **(G)**]. Paired *t*-tests, without multiple comparison correction, *p < 0.05, **p < 0.01, ns, not significant. Graphs represent mean ± SEM. Statistics for each test is reported in the Supplementary Table 4.

Flies with the genotype (*w, UAS-GCaMP3.0; +; Or22a-Gal4*) were anesthetized in ice for 30 min, and then placed into a truncated 1 ml pipette tip, leaving the head out of the tip and fixed using odor-free glue. The truncated tip was fixed using modeling clay on a custom Plexiglas mounting block (Strutz et al., 2012). Next, a custom holder to fix the antenna in vertical position was produced (Figures 1A–C and Supplementary Figure 1). A piece of aluminum foil (5 mm × 5 mm) with a slit (0.5 mm) for inserting the antenna and a small plastic ring (cut from an Eppendorf Microloader™) were glued on a glass coverslip (18 mm × 24 mm, #0 thickness, Menzel-Gläser, Braunschweig, Germany). The arista was then glued on the top of the glass coverslip with odor-free glue and the funiculus was manually cut at around half of its length with a scalpel blade #22 (Fine Science Tools, Heidelberg, Germany). Immediately after cutting the antenna, a small glass coverslip (15 mm × 15 mm, #00 thickness, Menzel-Gläser) moistened with a very thin layer of halocarbon oil 700 (Sigma-Aldrich) was laid on the open funiculus to seal it. Both coverslips were fixed to each other and to the Plexiglas holder with odor-free glue to prevent movement artifacts. Imaging was performed using a BX51W1 wide field fluorescence microscope (Olympus, Hamburg, Germany) equipped with a DCLP490 dichroic mirror and a 60x/0.90 water immersion LUMPFL objective (Olympus). The objective was immersed in a drop of distilled water put on top of the coverslip sealing the funiculus. GCaMP3.0 stimulation with a 475 nm light and an exposition time of 50 ms was performed using a monochromator (Polychrome V, TILL Photonics, Gräfelfing, Germany). Emitted light was filtered with a LP515 filter and acquired at a 4 Hz frequency using a cooled CCD camera (Sensicam, PCO Imaging, Kelheim, Germany) controlled by TILLVision 4.5 software (TILL Photonics). Odor stimuli were sampled from the headspace of a 50 ml volume glass bottle (Schott, Jena, Germany) containing 2 ml of ethyl hexanoate (99% purity, Sigma-Aldrich) diluted in mineral oil to a 10^–2^ dilution. A stimulus controller (Syntech, Kirchzarten, Germany) was used to deliver for 2 s the bottle headspace into a charcoal-filtered and humidified constant air flow (0.5 m/s) in a Teflon tube, where inlet was positioned 5–10 mm from the recorded antenna. The stimulus was delivered at 10 s from the start of the recording; the total duration of the experiment was 35 s. The response magnitude was calculated for each frame as the average ΔF/F_0_ and expressed in percentage after background subtraction. Regions of interest (ROIs) were selected using the built-in tools of TILLVision 4.5 and F_0_ was estimated as the mean fluorescence level calculated for each selected region of interest as the average intensity from 3 to 5.25 s of the recording and paired *t*-tests were performed using Prism 4 software (GraphPad Software Inc., La Jolla, CA, United States).

### Functional Calcium Imaging From an *ex vivo* Antenna Preparation

Four to eight old female flies of the genotype (*w; UAS-GCaMP6f; Orco-Gal4*), (*w; UAS-GCaMP6f; Or22a-Gal4*) were decapitated, then the antennae were excised and fixed on a Sylgard-coated support using the two-component silicon curing medium and immersed in *Drosophila* Ringer solution (130 mM NaCl, 5 mM KCl, 4 mM MgCl_2_ × 6H_2_O, 2 mM CaCl_2_, 36 mM sucrose, 5 mM Hepes, pH = 7.3, osmolality = 312 mOsm/kg). Samples were immersed in *Drosophila* Ringer solution and imaged using the imaging setup described above with a LUMPFL 60x/0.90 water immersion objective (Olympus, Hamburg, Germany). Emitted light was separated by a 490 nm dichroic mirror and filtered with a 515 nm long-pass filter. GCaMP6f was excited with a 475 nm light for 50 ms per frame and a temporal resolution of 0.2 Hz. Stimuli consisted of 100 μl of ethyl hexanoate (99%, Sigma-Aldrich) or VUAA1 (N-(4-ethylphenyl)-2-((4-ethyl-5-(3-pyridinyl)-4H- 1,2,4-triazol-3-yl)thio)acetamide, synthesized by the working group “Mass Spectrometry/Proteomics” of the Max Planck Institute for Chemical Ecology, Jena) at the required concentration. Ethyl hexanoate and VUAA1 working solutions were freshly prepared from 100 mM (or 500 mM, for final concentrations >100 μM) stocks in DMSO, kept at −20°C. DMSO at a 1:1000 dilution in *Drosophila* Ringer was used as control.

### Calcium Imaging and Data Analysis From *D. melanogaster* Olfactory Neurons

Females between 4 and 8 days old were decapitated, the antennae were excised and fixed on a Sylgard-coated support (Sylgard 184, Dow Corning Corp., Midland, MI, United States) using a two-component silicon curing medium (Kwik-Sil, World Precision Instruments, Sarasota, FL, United States). The antennae were immersed in dissecting solution (Au – Sicaeros et al., 2007) (Solution A: 137 mM NaCl, 5.4 mM KCl, 0.17 mM Na_2_HPO_4_, 0.22 mM KH_2_PO_4_. Solution B: 9.9 mM HEPES). For 500 ml of dissecting solution: 400 ml ultra-filtered water, 25 ml of Solution A, 14 ml of Solution B, 3.0 g (33.3 mM) D(+)-Glucose, 7.5 g (43.8 mM) Sucrose. Brought to pH 6.7 with 1 N NaOH and to the final volume of 500 ml with ultra-filtered water supplemented with 1 mM EDTA, 5 mM L-Cysteine hydrochloride (Cat. Nr. C1276, Sigma-Aldrich), and after equilibrating the pH to 6.7 with 1 N NaOH, 0.5 mg/ml Papain (Cat. Nr. 5125, Calbiochem, San Diego, CA, United States). Funiculi were cut between one third and one-half of their length and incubated at 27°C for 30 min. After incubation, the dissecting solution was removed and the antennae were rinsed twice for 5 min at 27°C with Ca^2+^-free Ringer (130 mM NaCl, 4 mM MgCl_2_ × 6H_2_O, 36 mM sucrose, 5 mM HEPES, 5 mM KCl. Osmolality = 312 mOsm/kg, pH = 6.7 adjusted with 1 N NaOH) supplemented with 1:75 cOmplete protease inhibitor cocktail (Cat. Nr. 04693116001, Roche, Basel, Switzerland) dissolved in a 100 mM PBS solution (50.93 mM Na_2_HPO_4_, 60.22 mM KH_2_PO_4_, 80.42 mM NaCl; pH = 6.7 adjusted with 1 N NaOH). A stock of Protease inhibitory solution was prepared by dissolving one tablet of cOmplete Protease Inhibitor Cocktail in 2 ml of 100 mM PBS. The solution was aliquoted and stored at −20°C for max 3 months. As an excessive concentration of cOmplete protease may cause cell permeabilization, the protease solution was added to the Ca^2+^-free Ringer in a 1:75 dilution, as suggested by the manufacturer.

### Glass Coverslip Preparation

Round glass coverslips (12 mm diameter, Cat. Nr. P231.1, Carl Roth, Karlsruhe, Germany) were cleaned by immersion in methanol (≥99.5%, Roth) and HCl (≥32%, Sigma-Aldrich) 1:1 for 30 min and after rinsing in double distilled water, by immersion in H_2_SO_4_ (95∼97%, Sigma-Aldrich) for 30 min (Cras et al., 1999). Coverslips were then thoroughly rinsed and kept in methanol under N_2_; immediately before use, they were thoroughly washed in double distilled water and dried under N_2_.

### Glass Capillaries and Dissociated Antennal Tissue Preparation

Borosilicate glass capillaries (0.86 mm × 150 mm × 80 mm, Cat. Nr. GB150-8P, Science Products, Hofheim, Germany) were pulled using a P-97 Micropipette puller (Sutter Instrument, Novato, CA, United States) and their tip was cut and fire polished in order to obtain holding micropipettes with an internal diameter ∼0.4 mm. The capillary tip size was found to be critical in order to extract viable neurons. The inner diameter of the tip should be slightly larger than the width of the cut antenna when fixed in a vertical position. The capillaries were subsequently silanized by immersion for 10–15 s in 5% dimethyldichlorosilane in toluene (Cat. Nr. 33065, Supelco, Sigma-Aldrich) and rinsed twice in toluene (≥99.5%, Sigma-Aldrich) and three times in methanol (≥99.5%, Sigma-Aldrich). The capillaries were then dried under N_2_ and heated at 200°C for 2 h.

To embed the dissociated antennal tissue, we first used 0.01% sterile filtered poly-L-lysine (Sigma-Aldrich, article no. P4707), Concanavalin A/laminin (Sigma-Aldrich: article no. C2010, L2020) or Collagenase Clostridium (Sigma-Aldrich, article no. C9891) on the coverslips to adhere cells but *Drosophila* OSNs did not attach to the cover slip. We next used a sodium metasilicate solution (Avnir et al., 2006; Pierre and Rigacci, 2011) to adhere the cells, for that, this solution was prepared immediately before use by mixing 2.71 μl of HCl 0.05 M, 2.43 μl of sodium metasilicate solution (Cat. Nr. 13729, Sigma-Aldrich) diluted 1:10 in double distilled water and 13.86 μl of low Ca^2+^-Schneider’s medium [Cat. Nr. S9895, Sigma-Aldrich, modified with 0.4 g EGTA (1 mM), 22.2 mg CaCl_2_ (free Ca^2+^ = 500 nM), 0.4 g NaHCO_3_, total volume: 1 L, pH = 6.7 adjusted with 1 N NaOH; sterile filtered and kept at 4°C]. After a 1 μl drop of Ca^2+^-free Ringer was deposited on a cleaned coverslip, the content of the treated antennae was gently sucked using a silanized capillary attached to a 2 μl micropipette. Usually ∼0.5/1 μl of liquid was sucked together with each antenna. Within 10 min from mixing, the complete volume of the silicate gel solution was added to the coverslip and distributed uniformly. The total volume of liquid on a coverslip should be ∼ 25 μl, for a final Na_2_SiO_3_ concentration equal to 0.972% of the Na_2_SiO_3_ (≥27% SiO_2_ basis) stock solution. Coverslips were incubated for 1 h at 26°C in a high humidity environment, to avoid desiccation.

### Data Analysis

Imaging data were exported as uncompressed tiff files and analyzed using custom scripts in Fiji-ImageJ2 (Schindelin et al., 2012; Rueden et al., 2017) where the regions of interest were selected using a semi-automatic procedure and the ΔF/F_0_ values were calculated after background, flat-field and movement correction. Statistical analysis was performed in R (R Core Team, 2017) using custom scripts including add-on packages (Chang, 2014; Ritz et al., 2015; Wickham, 2016; Arnold, 2017; Auguie, 2017). Parametric statistics for data analysis of the standard deviation of base level ΔF/F_0_ values for GCaMP6f (Figure 3I) was used after evaluation of the pooled ΔF/F_0_ values distribution of all analyzed cells (ROIs) (Supplementary Figure 5 and see section “Statistical Methods” below). In all preparation types we occasionally observed a reduction in base level fluorescence (e.g., Figure 1 and Supplementary Figure 5). Such artifacts, e.g., due to fluophore bleaching were compensated during data analysis.

### Statistical Methods

The appropriate statistics for data analysis on Supplementary Figures 2D,E and Figure 3I was evaluated after assessment of the data distributions. For GCaMP6f ΔF/F_0_ data relative frequency distributions − including all regions of interest (ROIs) selected for each treatment− were evaluated for the basal intracellular free Ca^2+^ concentration ([Ca^2+^]_*i*_) at time *t* = 0 s (Supplementary Figure 4). Moreover, the standard deviation of ΔF/F_0_ values before stimulus application was calculated for each ROI and the relative frequency distribution of values for each treatment was assessed (Supplementary Figure 5). In this case, none of the treatments showed a terminal long tail, meaning that the number of ROIs with skewed high values − and consequently with the chance to skew the mean − was negligible. Consequently, the mean value between all ROIs was selected for each independent measure and the difference between groups was evaluated using two-tailed Welch’s *t*-tests.

## Results

### *In vivo* Cellular-Resolution Calcium Imaging From Antennal Olfactory Sensory Neurons

The isolated antenna preparation according to Mukunda et al. (2014) allows to study OR activation in the OSNs. This approach, however, requires dissolved OR activators. To overcome this limitation we developed a whole animal preparation that allows to stimulate OSNs with airborne odorants. To access the antennal olfactory neurons in a living fly, we first immobilized the animal in a custom Plexiglas holder inside a pipette tip and we fixed the antenna in vertical position using a custom holder without disrupting the antennal sensilla (Figures 1A,B and Supplementary Figure 1). After that, we made a transversal cut of the funiculus, stimulated the animal with airborne odorants, and recorded their neuronal activity (Figures 1C–E). The transversal cut did not induce disruption of the antennal tissue sufficient to impair the ability of olfactory neurons to respond to odors. Using a fly line expressing GCaMP3.0 in Or22a olfactory neurons we checked for the viability of these cells by recording their responses first to mineral oil (solvent) and comparing them to the responses elicited by ethyl hexanoate at a 10^–2^ dilution in mineral oil. We could show the viability of this preparation as cells were responding to a 10^–2^ dilution of the odor, but not to the solvent alone (Figures 1F,G). Moreover, the response was sustained and lasted up to 20 s after stimulation (Figures 1H,I and Supplementary Table 4).

### *In vitro* Cellular-Resolution Calcium Imaging From Dissociated Antennal Tissue

**FIGURE 2:**
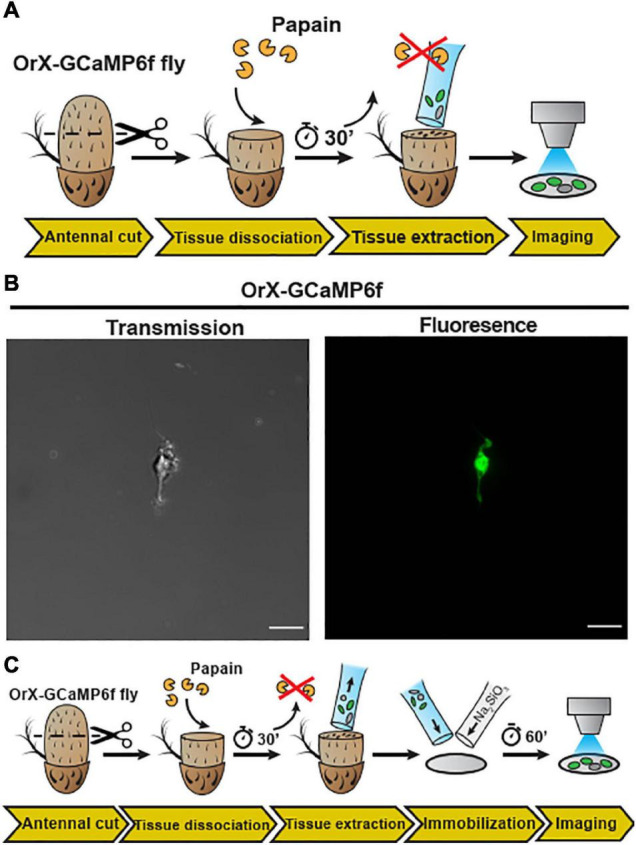
Embedding of dissociated *D. melanogaster* antennal tissue in a sodium metasilicate gel. **(A)** Schematic procedure for dissociation and embedding of vinegar fly antennal tissue (see section “Materials and Methods”). After fixing the antenna with a silicon-based curing medium, the funiculus was cut and incubated with a papain solution for 30 min. Then, the dissociated tissue was extracted with a silanized glass capillary and then single neuron was extracted from this fly (*+; Orco-Gal4/CyO; UAS-Syn21-GFP-p10/TM6B*), using this preparation. **(B)** Confocal image of single cell as shown in transmission (gray) and fluorescence (green) signals, Scale bar = 8 μm. In the next illustration **(C)**, after tissue extraction, the sample was mixed with a modified *Drosophila* Schneider’s medium containing 0.972% of a Na_2_SiO_3_ stock solution (≥27% SiO_2_ basis) on a methanol treated cover slip. Ca^2+^ imaging was performed after a 60 min incubation time, to allow the Na_2_SiO_3_ gelation.

With the whole animal preparation and open antenna preparation, calcium imaging on *Drosophila* OSNs can be studied under *in situ* conditions. However, to apply high-resolution techniques such as cryogenic electron microscopy (Cryo-EM) or direct stochastic optical reconstruction microscopy (dSTORM; Heilemann et al., 2008) imaging requires single *Drosophila* OSNs. We thus established a protocol to mildly digest *Drosophila* antennal tissue and then isolate OSNs. In detail, after funiculi from excised antennae were transversally cut, the antennal tissue was partially digested with papain − allowing the extraction of the digested content using silanized glass capillary (see schematic diagram Figure 2A). A drop of low Ca^2+^ Schneider’s medium containing *Drosophila* OSNs from (*+; Orco-Gal4/CyO; UAS-Syn21-GFP-p10/TM6B*) fly line were kept on a glass slide, covered with a cover slip and then single cell was immediately captured using laser scanning microscopy [Figure 2B, transmission (left), fluorescence (right)]. Next, in order to perform physiological recordings from dissociated tissue, it was necessary to adhere the cells. Initially, we used standard histology procedures to adhere the isolated OSNs on to the coverslip by coating it with Poly-L-lysine, Collagenase Clostridium, or Concanavalin A/Laminin but we failed to attach the cells using this approach.

We thus established a sodium metasilicate hydrogel system to embed biological tissue in hope to successfully apply this embedding system for the isolated *Drosophila* OSNs. First we started by assessing the effects of the gelation process on the properties of pH-buffered solutions. We found that Na_2_SiO_3_ had significantly smaller effects on the buffer osmolality than other embedding media as agarose and sodium alginate (Supplementary Figure 2B) and this parameter was not affected by the silicate polymerization process (Supplementary Figures 2C, 3), making sodium silicate hydrogels suitable for functional studies. In particular, sodium metasilicate (Na_2_SiO_3_)-based aqueous solutions constitute an interesting case as they polymerize through a pH-driven condensation reaction (Pierre and Rigacci, 2011) with salts and water as the only byproducts. Partial neutralization of basic Na_2_SiO_3_ solutions (pH ∼ 12.5) to physiological (pH ∼ 6.7–7.4) pH levels induce the gelation of colloidal silica particles through a chain of condensation reactions (Rao et al., 2011; Supplementary Figure 2A).


$$Na_{2}SiO_{3}+2HCl+\left(x-1\right)H_{2}O\to SiO_{2}\cdot xH_{2}O+2NaCl$$

**FIGURE 3:**
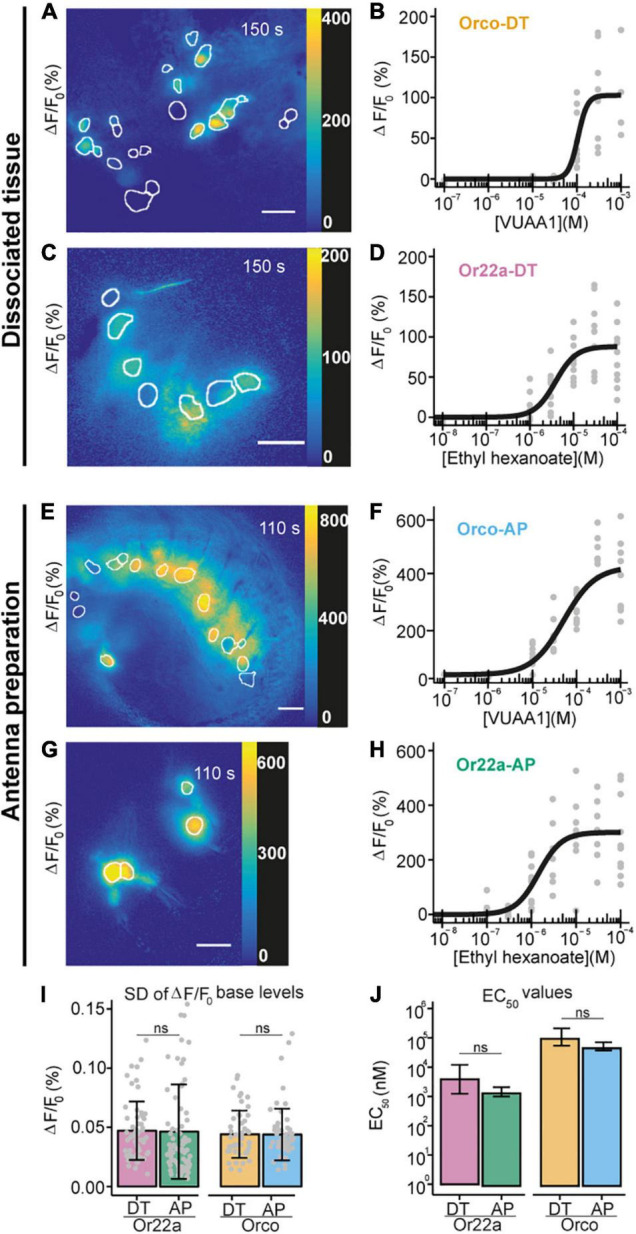
Comparison between Ca^2+^ imaging from OSNs in sodium metasilicate-embedded and undissociated antennal tissue. **(A–D)** ΔF/F_0_ (%) from antennal dissociated tissue with **(A)** Orco expressing neurons and **(C)** Or22a OSNs following the application of ethyl hexanoate (EH) and VUAA1, respectively, at dose-dependent concentration. White boundaries indicate regions of interest (ROIs) for quantitative analysis. Gray lines show the ΔF/F_0_ (%) of each ROI shown in the corresponding left panel; the black line represents the average taken for subsequent analysis (*n* = 1). Scale bar = 8 μm. **(B,D)** Concentration-response curves for Na_2_SiO_3_-embedded dissociated antennal tissue with Orco (**B**, Orco-DT) and Or22a OSNs (**D**, Or22a-DT) stimulated with VUAA1 (4 ≤ *n* ≤ 11 for each concentration, 49 total data points) and ethyl hexanoate (8 ≤ *n* ≤ 10 for each concentration, 60 total data points), respectively. Examples of Ca^2+^ imaging from Orco **(E)** and Or22a-expressing **(G)** OSNs from undissociated antennal tissue with the ROIs highlighted in white. **(F,H)** Concentration-response curves from Orco **(F)** and Or22a-expressing OSNs **(H)** from undissociated antennal tissue after stimulation with increasing concentrations of VUAA1 (Orco-AP) **(F)** and ethyl hexanoate (Or22a-AP) **(H)**. 5 ≤ *n* ≤ 11 **(F)** and 6 ≤ *n* ≤ 11 **(H)** for each concentration, 60 and 71 total data points, respectively. Parameters for curve fits are reported in Supplementary Table 5. **(I,J)** Comparison between the ΔF/F_0_ standard deviation (SD) before stimulus application **(I)** and the half maximal effective concentrations (EC_50_) **(J)** of the OSNs from theundissociated antennal preparation (AP) and the dissociated tissue (DT) after stimulation of Or22a OSNs with ethyl hexanoate and Orco OSNs with VUAA1. **(I)** Or22a-AP: *n* = 71, Or22a-DN: *n* = 60, Or22a AP vs. DT: *p* = 0.9036. Orco-AP: *n* = 60, Orco-DN: *n* = 49, Orco AP vs. DT: *p* = 0.9816. Two-tailed Welch two-sample t-tests. Graphs represent mean ± SD. Test statistic values, confidence intervals and degrees of freedom are given in Supplementary Table 6. **(J)** Or22a AP vs. DT: *p* = 0.1313. Orco AP vs. DT: *p* = 0.06676. Parameter comparison using the compParm function of the R (R Core Team, 2017) drc (Ritz et al., 2015) package. Statistic values are given in Supplementary Table 7. Graphs represent mean ± 95% CI.

We then explored the effects of Na_2_SiO_3_ on the dynamics of the morphology and the free intracellular calcium concentration ([Ca^2+^]_*i*_) of adherent HEK293 cells (Supplementary Figures 2D–F). Calcium imaging using the dye Fura2-AM demonstrated that Na_2_SiO_3_ did not affect the cell basal [Ca^2+^]_*i*_ (Supplementary Figure 2D) and did not induce variations of the [Ca^2+^]_*i*_ over time (Supplementary Figure 2E). Moreover, we observed changes in the cell morphology due to Na_2_SiO_3_ only at high concentrations (>1.5% of a ≥27% SiO_2_ basis stock solution, see section “Materials and Methods”), where cells tended to aggregate in clusters (Supplementary Figure 2F). We can conclude that Na_2_SiO_3_ hydrogels are compatible with the embedding of living cells for functional studies. Therefore, we now dissociated *Drosophila* antennal tissue and then embedded on a clean glass coverslip using a modified Schneider’s medium containing 0.972% Na_2_SiO_3_ stock solution (see schematic diagram Figure 2C). This concentration of Na_2_SiO_3_ allowed us to reliably embed the tissue samples while having minimal effects on the cell physiology (Supplementary Figure 3). Following this procedure, we obtained OSNs that retained their morphology, including the ciliated outer dendritic segment (Figures 3A,C). The density of isolated OSNs was about 20 OSNs/100 μm^2^.

We then asked whether the embedded neurons also retained their functional properties. We performed Ca^2+^ imaging from OSNs expressing the calcium indicator GCaMP6f (Chen et al., 2013) under the co-receptor Orco or the odorant receptor Or22a promoters and stimulated OSNs expressing Orco or Or22a with their respective agonists, the synthetic compound VUAA1 (Jones et al., 2011; Figure 3A) and ethyl hexanoate (Münch and Galizia, 2016; Figure 3C and Supplementary Video 1). After extraction of regions of interest (see section “Materials and Methods”), we calculated the changes in GCaMP6f fluorescence intensity with respect to the base level expressed in percent (% ΔF/F_0_). In both cases, OR agonists induced calcium responses in a concentration-dependent manner (Figures 3B,D). In order to determine if the OSN response profile was affected by the tissue dissociation process or Na_2_SiO_3_, we performed calcium imaging from excised antennae immediately after the funiculus cut (Figures 3E–H), and we compared the response profiles of Orco and Or22a-expressing OSNs from dissociated and undigested antennal tissue (Figures 3I,J). The response profiles between the OSNs in dissociated and undigested tissues showed differences in the maximal intensity and time course of Ca^2+^ responses (Supplementary Figure 5). Nevertheless, we did not find significant differences in the fluctuation of the basal fluorescence levels (Figure 3I) and in the EC_50_ of concentration-response curves for Or22a neurons stimulated with ethyl hexanoate and Orco expressing neurons stimulated with VUAA1 (Figure 3J). In addition to the excitatory ethyl hexanoate, we tested the inhibitory benzaldehyde. At 10^–3^ dilution we observed a decrease in GCaMP6f fluorescence intensity ΔF/F_0_ by 7 ± 4% (*n* = 6) (Supplementary Figure 7). This illustrates that after cell isolation and embedding the inhibitory response remained conserved, as observed upon heterologous expression of Or22a (Wicher et al., 2008). OSN isolation removes the direct contact of these neurons to their support cells. Ablation of thecogen cells which directly envelop OSNs did not affect the odor selectivity of tested cells (Prelic et al., 2022).

This suggests that the tissue dissociation and embedding procedures, at the Na_2_SiO_3_ concentration used, did not significantly affect the viability of OSNs as well as the diffusion of the OR agonists through the embedding medium.

## Discussion

We here report the development and the validation of new preparations to access antennal OSNs and to perform functional imaging experiments in *in vivo and* dissociated tissue conditions. As olfactory stimuli are usually carried by air, studies of olfactory function using water-borne stimuli may lead to artifacts, due to the much higher time scale in which these experiments are usually carried and the irreversible change of the sensillum and antennal fluids to a standard Ringer solution. Therefore, we designed an *in vivo* preparation to study the activity of antennal OSNs at cellular resolution under more natural conditions. We could obtain Ca^2+^ imaging responses from olfactory neurons expressing the Or22a receptor, after stimulation with the agonist ethyl hexanoate, but not with the mineral oil solvent (Figure 1). This preparation is most suited for experiments, which require delivering odors through an airstream with higher temporal resolution, or where it is important to keep unaltered the composition of the antennal and sensillum fluids. By combining it with the genetic tools available in *Drosophila*, e.g., RNA interference, such technique can be of great help to investigate signal transduction mechanisms of insect olfactory neurons in their native environment. Thus, this *in vivo* preparation fills the gap between techniques that allow odor stimulation via air but getting no spatially resolved output like single sensillum recordings and those allowing to observe odor response on OSN level but with stimuli provided via solution. Our finding that the odor responses observed with the *in vivo* preparation do not qualitatively differ from those obtained in the previous open antenna preparation supports the view that both approaches provide consistent results.

We next isolated *Drosophila* OSNs and embedded them in a permeable watery-based medium sufficiently rigid to allow the fixation of the preparation, but at the same time allowing the stimulus to penetrate through it. Remarkably, such medium did not require any sort of heating making it more suitable than low temperature-melting agarose media, which tended to damage the neurons and compromise functional imaging experiments (Figure 3 and Supplementary Figure 2B). Here, the gelation process is driven by a series of hydrolysis and condensation reactions happening at room temperature and with the fastest kinetics at pH ∼ 7 (Pierre and Rigacci, 2011), making this method extremely attractive for applications in physiology and neuroscience. Moreover, the use of silica aero- and hydro-gels is already established in biotechnology to build reactors for the encapsulation of DNA molecules, enzymes and bacteria to accelerate biochemical reactions (Gill and Ballesteros, 2000; Depagne et al., 2011) or to design ceramics for medical applications (Vallet-Regí, 2001). We here demonstrated for the first time − to our knowledge − that sodium metasilicate hydrogels are excellent cell and tissue embedding agents for imaging experiments as they stabilize samples on uncoated glass surfaces, while retaining the function of neural cells without appreciable signs of cellular stress. The absence of toxic byproducts, the ability to form the gel at room temperature, together with the colloidal organization of silicate particles, which does not interfere with the osmolality of saline solutions during the gelation process, and its complete transparency make Na_2_SiO_3_ hydrogels an attractive choice, when embedding neural cell and tissue samples for physiological investigations.

This preparation maintains the native cellular environment of the ORs which is in contrast to a heterologous expression system or even in an empty neuron system (Fleischer et al., 2018). In the latter one, although the ORs are expressed in OSNs, the sensillar composition might be less compatible with the OR (Fleischer et al., 2018). On the other hand, the isolated OSN preparation lacks the surrounding support cells present in the native tissue. As shown in Prelic et al. (2022), these support cells contribute to the management of ion homeostasis. This might be one reason that we observe differences in the odor response size and time course between isolated OSNs and OSNs in the antenna preparation (Supplementary Figure 5).

As the isolated OSN preparation is an acute procedure, there are no changes in the protein expression level which can be observed in long term cell cultures. In cockroach neurons occurred, for example, a neosynthesis of Na^+^ channels after 24 h culture (Tribut et al., 1991).

## Conclusion

These two new preparations described here form the basis for the development of new tools to access and to investigate OSNs under controlled conditions. This is much of a necessity in order to validate studies based on the expression of insect proteins in heterologous systems and to advance our knowledge of olfactory signal transduction in insects.

## Data Availability Statement

The original contributions presented in the study are included in the article/Supplementary Material, further inquiries can be directed to the corresponding author.

## Author Contributions

DW, FM, and BH formulated the ideas for the study. FM and DW designed the study. JB contributed to study design. KJ contributed to Figure 2B and Supplementary Figure 7. FM and SK performed the experiments. FM analyzed the data. FM, KJ, and DW wrote the manuscript. All authors contributed to the final version of the manuscript.

## Conflict of Interest

The authors declare that the research was conducted in the absence of any commercial or financial relationships that could be construed as a potential conflict of interest.

## Publisher’s Note

All claims expressed in this article are solely those of the authors and do not necessarily represent those of their affiliated organizations, or those of the publisher, the editors and the reviewers. Any product that may be evaluated in this article, or claim that may be made by its manufacturer, is not guaranteed or endorsed by the publisher.
