# Supplementary material for: Targeting Insect Olfaction in vivo and in vitro Using Functional Imaging
Source: Front Cell Neurosci. 2022 Feb 24;16:839811. doi: 10.3389/fncel.2022.839811 (PMC8907589; doi:10.3389/fncel.2022.839811)
Supplement: Supplementary file 1 [file Data_Sheet_1.pdf]

Targeting insect olfaction *in vivo* and *in vitro* using functional imaging

Fabio Miazzi[1], Kalpana Jain[1], Sabine Kaltofen[1], Jan E. Bello[2], Bill S. Hansson[1,†] and Dieter Wicher[1†,*]

[1] Max Planck Institute for Chemical Ecology, Department of Evolutionary Neuroethology, Hans-Knoell Str. 8, D-07745 Jena, Germany.
[2] University of California – Riverside, Department of Entomology,
3401 Watkins Dr., Riverside, CA 92521.
[†] These authors shared supervision

*** Correspondence:**
Dieter Wicher
dwicher@ice.mpg.de

Supplementary Methods

Sodium metasilicate solution titration

A sodium metasilicate stock solution (≥ 27% SiO_2 basis and ≥ 10% NaOH, Cat. Nr. 13729, Sigma-Aldrich, Steinheim, Germany) was first titrated in a 1:1000 dilution in double distilled water with 0.01 N HCl and pH changes were recorded with a pH meter. The fit of the titration curve and the inflection point were calculated using CurTiPot (Gutz, I. G. R., CurTiPot – pH and Acid-Base Titration Curves: Analysis and Simulation software, version 4.2. http://www.iq.usp.br/gutz/Curtipot_.html). The stock sodium silicate solution had a $[OH^-]$ = 0.558 M.

HEK293 cells culture and imaging

HEK293 cells (DSMZ Nr. ACC 305) were purchased from the Leibniz Institute DSMZ GmbH (Braunschweig, Germany) and grown in DMEM/F12 1:1 medium (Cat. Nr. 11320, Gibco, Life Technologies, Grand Island, NY, USA) supplied with 10% Fetal Bovine Serum at 37°C and 5% CO_2. For experiments 80-90% confluent cells were dissociated by trypsinization and were subsequently cultured on poly-l-lysine (0.01%, Sigma-Aldrich, Steinheim, Germany) coated glass coverslips (12 mm diameter, Cat. Nr. P231.1, Carl Roth, Karlsruhe, Germany) at ~1×10^5cells/well in a 24 wells plate with DMEM/F12 1:1 medium with 10% Fetal Bovine Serum at 37°C and 5% CO_2. 24 hours post-plating, cells were incubated in Opti-MEM medium (Cat. Nr. 31985, Gibco), containing 5 µM Fura-2 acetoxymethyl ester (Fura2-AM; Molecular Probes, Invitrogen, Carlsbad, CA, USA) for 30 min at 37°C and 5% CO_2. Then cells were washed twice in 26°C pre-warmed modified Hepes-HBSS (131.63 mM NaCl, 5.3 mM KCl, 0.5 mM $MgCl_2 \times 6H_2O$, 1 mM EGTA, 0.2 mM $CaCl_2$, 0.4 mM $MgSO_4$, 0.44 mM KH_2PO_4, 4.17 mM $NaHCO_3$, 0.34 mM Na_2HPO_4, 2 mM D-Glucose, 20 mM Hepes, pH = 7.4, osmolality = 296 mOsm/Kg) and 50 µl of modified Hepes-HBSS alone (Control) or with a sodium metasilicate (Na_2SiO_3) stock solution (≥ 27% SiO_2 basis and ≥ 10% NaOH, Sigma-Aldrich) diluted 1:10 in double distilled water was applied on each coverslip (solutions: (i) 0.5% Na_2SiO_3 stock: 2.79 µl of HCl 0.05 M, 2.5 µl of Na_2SiO_3 diluted 1:10, 44.71 µl of modified Hepes-HBSS; (ii) 1% Na_2SiO_3 stock: 5.58 µl

of HCl 0.05 M, 5 µl of Na_2SiO_3 diluted 1:10, 39.42 µl of modified Hepes-HBSS; (iii) 1.5% Na_2SiO_3 stock: 8.36 µl of HCl 0.05 M, 7.5 µl Na_2SiO_3 diluted 1:10, 34.14 µl of modified Hepes-HBSS; (iv) 2% Na_2SiO_3 stock: 11.15 µl of HCl 0.05 M, 10 µl Na_2SiO_3 diluted 1:10, 27.85 µl of modified Hepes-HBSS). Cells were incubated for one hour at 26°C in a humidified incubator to avoid desiccation. After incubation cells were kept throughout the experiment in standard extracellular solution (SES) containing 135 mM NaCl, 5 mM KCl, 1 mM $CaCl_2$, 1 mM $MgCl_2 \times 6H_2O$, 10 mM HEPES, 10 mM glucose (pH = 7.4; osmolality = 295 mOsmol/Kg). Excitation with 340 and 380 nm light was obtained using a monochromator (Polychrome V, TILL Photonics, Gräfelfing, Germany), coupled to an epifluorescence microscope (Axioskop FS, Zeiss, Jena, Germany) by means of a water immersion objective (LUMPFL40×W/IR/0.8; Olympus, Hamburg, Germany) and controlled by an imaging control unit (ICU, TILL Photonics). Emitted light was separated by a 400 nm dichroic mirror, filtered with a 420 nm long-pass filter and acquired by a cooled CCD camera (Sensicam, PCO Imaging, Kelheim, Germany) controlled by TILLVision 4.5 software (TILL Photonics). The exposure time was 150 ms per frame, with a temporal resolution of 0.2 Hz; in total each experiment lasted 50 s. The final image resolution was 640 × 480 pixels in a frame of 175 × 130 µm. Free intracellular Ca^{2+} concentration ($[Ca^{2+}]_i$) was calculated after background correction according to the equation:

$$[Ca^{2+}]_i = K_{eff}\frac{R - R_{min}}{R_{max} - R}$$

Where K_{eff} = 1.95 µM, R_{min} = 0.2 and R_{max} = 5.3. Regions of interest (ROIs) were selected using the built-in tools of TILL Vision and saved as comma-separated values (csv) files. Data analysis was performed using R (R Core Team, 2017) including add-on packages (Chang, 2014; Wickham, 2016; Auguie, 2017; Arnold, 2017). Non parametric statistics for HEK293 data analysis was used after evaluation of the pooled $[Ca^{2+}]_i$ distribution of all analyzed cells (ROIs). As the $[Ca^{2+}]_i$ distributions of the parameters analyzed in Supplementary Figure (1b-c, 3), show a long-tailed distribution, the median value for each independent replicate was used for subsequent non-parametric statistics (see the "Statistical methods" section below).

For HEK293 cells relative frequency distributions – including all regions of interest (ROIs) selected for each treatment – were evaluated for the basal intracellular free Ca^{2+} concentration ($[Ca^{2+}]_i$) at time t =

0 s (Supplementary Fig. 3a) and the standard deviation of the $[Ca^{2+}]_i$ during the 50s time series imaging experiments (Supplementary Fig. 3b). In both cases, multiple treatments showed a distribution with a terminal long tail. Consequently, extreme values of few cells can skew the mean value of each independent sample. For this reason, non-parametric statistics was applied to the HEK293 dataset: the median value between all ROIs was evaluated for each independent sample and comparisons between groups were performed using two tailed Wilcoxon-Mann-Whitney tests with Holm's multiple test correction

Additional embedding media

Low melting temperature agarose (Cat. Nr. 6351, Roth) and sodium alginate (Cat. Nr. W201502, Sigma-Aldrich) in modified Hepes-HBSS at the desired concentration (% m/v) were incubated at 60 degrees until dissolved. Agarose solutions were cooled to 38 degrees before testing their osmolality, while sodium alginate solutions were cooled to room temperature and were tested without prior addition of calcium salts to induce their gelation. Osmolality of all solutions was tested with a Micro Osmometer 210 (Fiske, Norwood, MA, USA).

Supplementary figures

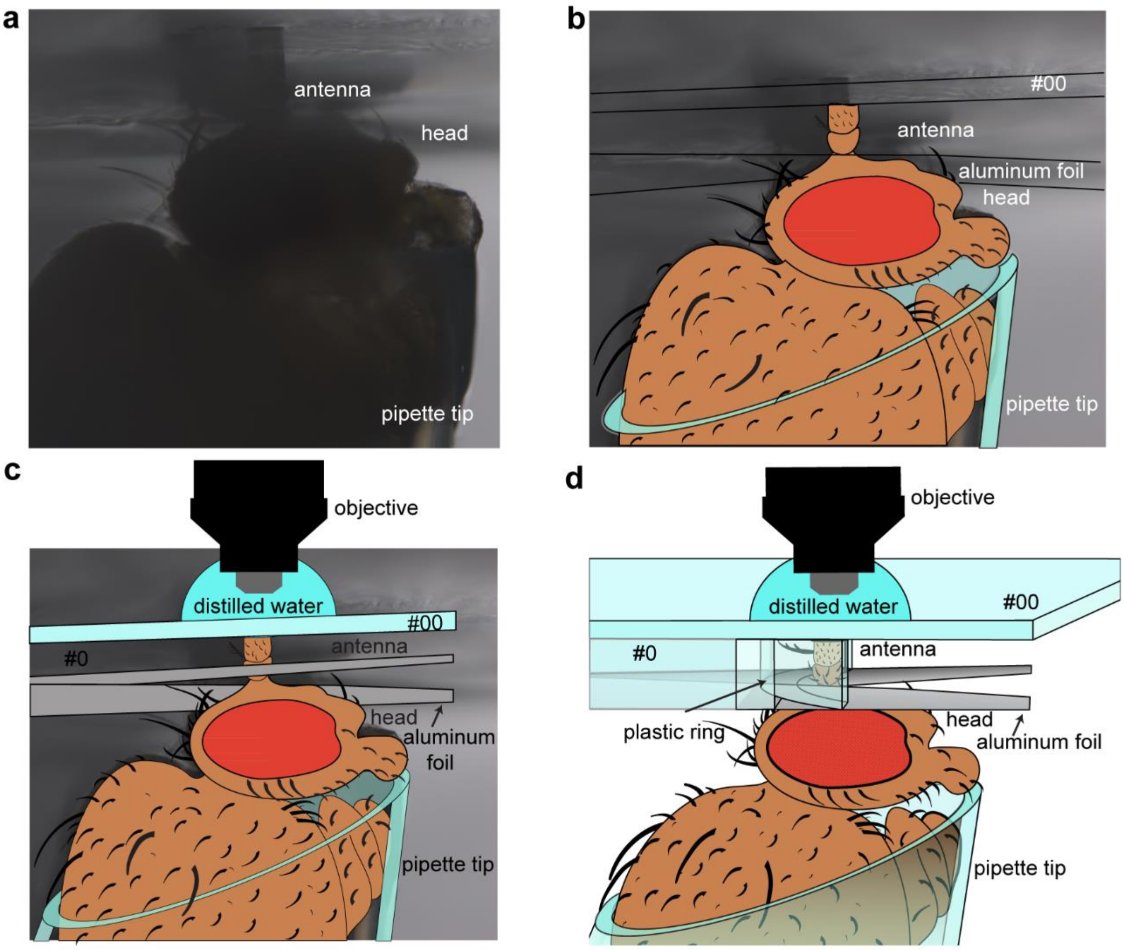

Supplementary Figure 1 | Sequential representation of the in vivo preparation.

(a) Image of the in vivo preparation from the side view. **(a - d)** The fly is placed in a 1ml truncated pipette tip fixed with an odor free glue and the antenna is held in vertical position on a custom holder, black line on the top represents #00 glass cover slip and on the bottom aluminum foil **(b).** The antenna is placed within the thickness of a #0 glass coverslip with the help of aluminum foil with a slit **(c)** and a small plastic ring **(d)**. After the tip of the funiculus is cut with a scalpel blade, a thin #00 glass coverslip moistened with halocarbon 700 oil is placed on top in order to seal the open antenna without soaking the sensilla **(d)** and also (shown in Figure 1c-d). BX51W1 wide field fluorescence microscope is used to image Or22a GCaMP3 neurons **(d)**.

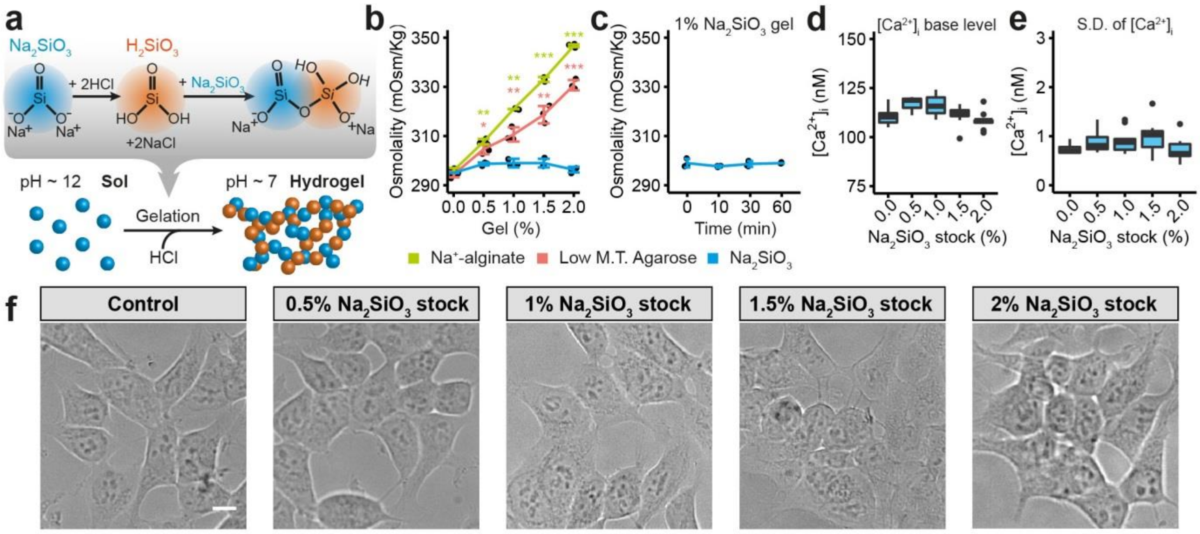

Supplementary Figure 2 | Sodium metasilicate (Na_2SiO_3) as an embedding agent for biological samples.

(a) Schematic representation of the silicate gelation in pH buffered solutions. Partial neutralization of basic Na_2SiO_3 solutions with an acid produce silicic acid (H_2SiO_3) molecules that react with other Na_2SiO_3 or H_2SiO_3 molecules to form silicate colloidal particles. On a macroscopic scale condensation around these silicate particles lead to the formation of a silicate-based hydrogel (Rao et al., 2011). **(b)** Effect of common gelation agents and Na_2SiO_3 on the osmolality of a modified Hepes-HBSS buffered solution (see Methods section). Sodium alginate and low melting temperature (M. T.) agarose induce a concentration-dependent change in the medium osmolality that is significantly higher than Na_2SiO_3 colloidal particles. Na^{2+}-alginate vs Na_2SiO_3: 0%: p = 0.4508; 0.5%, p = 0.002686; 1%, p = 0.00412; 1.5%, p = 0.00010528; 2%, p = 1.7388e-05. Agarose vs. Na_2SiO_3: 0%, p = 0.4508; 0.5%, p = 0.030430; 1%, p = 0.00718; 1.5%, p = 0.00355300; 2%, p = 1.0570e-04. Graph represents mean ± S.D., n = 3 for each concentration, two-tailed Welch's t-tests with Holm's multiple correction test. **(c)** Na_2SiO_3 polymerization does not affect the Hepes-HBSS buffer osmolality. Buffer osmolality was tested immediately or 10, 30 and 60 minutes after mixing the Na_2SiO_3, diluted HCl and Hepes-HBSS solutions (see supplementary methods section). 0 vs 10 min: p = 0.9399, 0 vs. 30 min: p = 1, 0 vs. 60 min: p = 1. Graphs represents mean ± S.D., n = 3 for each concentration, two-tailed Welch's t-tests with Holm's multiple correction test. **(d-e)** Box and whiskers plots representing **(d)** the basal $[Ca^{2+}]_i$ levels at t = 0 and **(e)** the standard deviation (SD) of $[Ca^{2+}]_i$ fluctuations of Fura2-AM loaded HEK293 cells incubated

in buffer alone (0%) or containing increasing concentrations of Na_2SiO_3. n = 8 for 0% and 1.5% Na_2SiO_3 stock; n = 7 for 0.5% Na_2SiO_3 stock; n = 9 for 2% Na_2SiO_3 stock; n = 10 for 1% Na_2SiO_3 stock. **(d)** 0% vs 0.5%: p = 0.05596, 0% vs. 1%: p = 0.07995, 0% vs. 1.5%: p = 0.65640, 0% vs. 2%: p = 0.65640. **(e)** 0% vs 0.5%: p = 0.21633, 0% vs. 1%: p = 0.34560, 0% vs. 1.5%: p = 0.19952, 0% vs. 2%: p = 0.48070. Two-tailed Wilkoxon-Mann-Whitney tests with Holm's multiple test correction (see supplementary methods section and Supplementary Fig. 3). Test statistic values, confidence intervals, degrees of freedom and uncorrected p values for panels **(b-e)** are reported in Supplementary Tables 1-3. * $p < 0.05$, ** $p < 0.01$, *** $p < 0.001$. **(f)** Morphology of HEK293 cells after incubation for one hour in presence of buffer alone (Control) or with increasing concentrations of sodium metasilicate (Na_2SiO_3). Cells tend to aggregate in clusters after incubation in presence of high Na_2SiO_3 concentrations (>1.5% Na_2SiO_3 stock, see online methods). Scale bar = 10 µm.

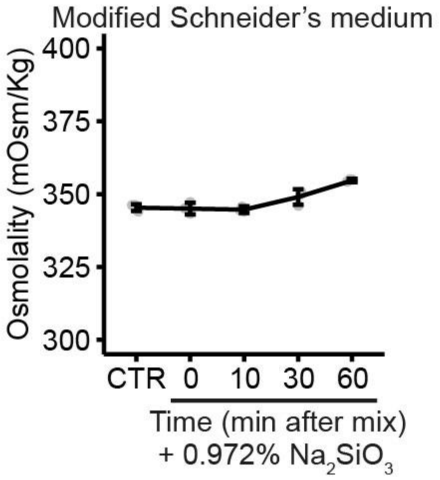

Supplementary Figure 3 | Osmolality changes in the modified Schneider's medium-based sodium metasilicate gel during polymerization.

Changes in osmolality during polymerization of the modified Schneider's medium used to embed *Drosophila* olfactory sensory neurons (as shown in Fig. 3) (CTR, buffer alone) with 0.972% of the Na_2SiO_3 stock solution (≥ 27% SiO_2 basis), measured immediately after mixing the components (0 min) or after 10, 30 and 60 min. 0 min vs. 10 min: p =1, 0 min vs. 30 min: p = 1, 0 min vs. 60 min: 0.3714. Two-tailed Welch's t-tests with Holm's multiple test correction. Graphs show mean ± SD and individual data points. n = 3 for each treatment.

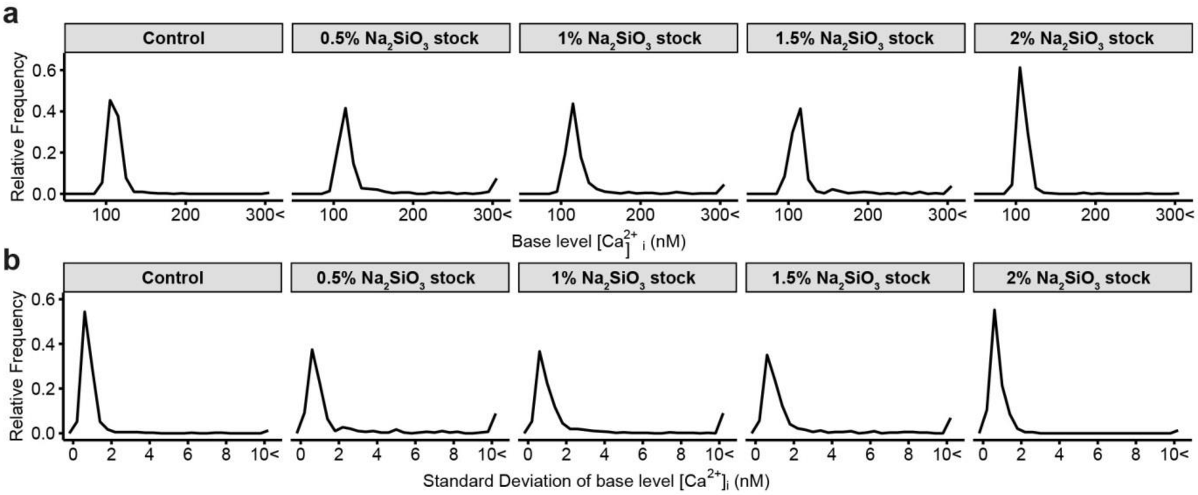

Supplementary Figure 4 | Distributions of analyzed parameters for HEK293 cells ROIs.

Relative frequency distribution of $[Ca^{2+}]_i$ base levels at t = 0 s **(a)** and standard deviation of $[Ca^{2+}]_i$ base levels over the 50 s time course **(b)** of all pooled HEK293 cells (as ROIs) for each treatment. Control: 403 ROIs from 8 independent replicates; 0.5% Na_2SiO_3: 294 ROIs from 7 independent replicates; 1% Na_2SiO_3: 467 ROIs from 10 independent replicates; 1.5% Na_2SiO_3: 319 ROIs from 8 independent replicates; 2% Na_2SiO_3: 384 ROIs from 9 independent replicates.

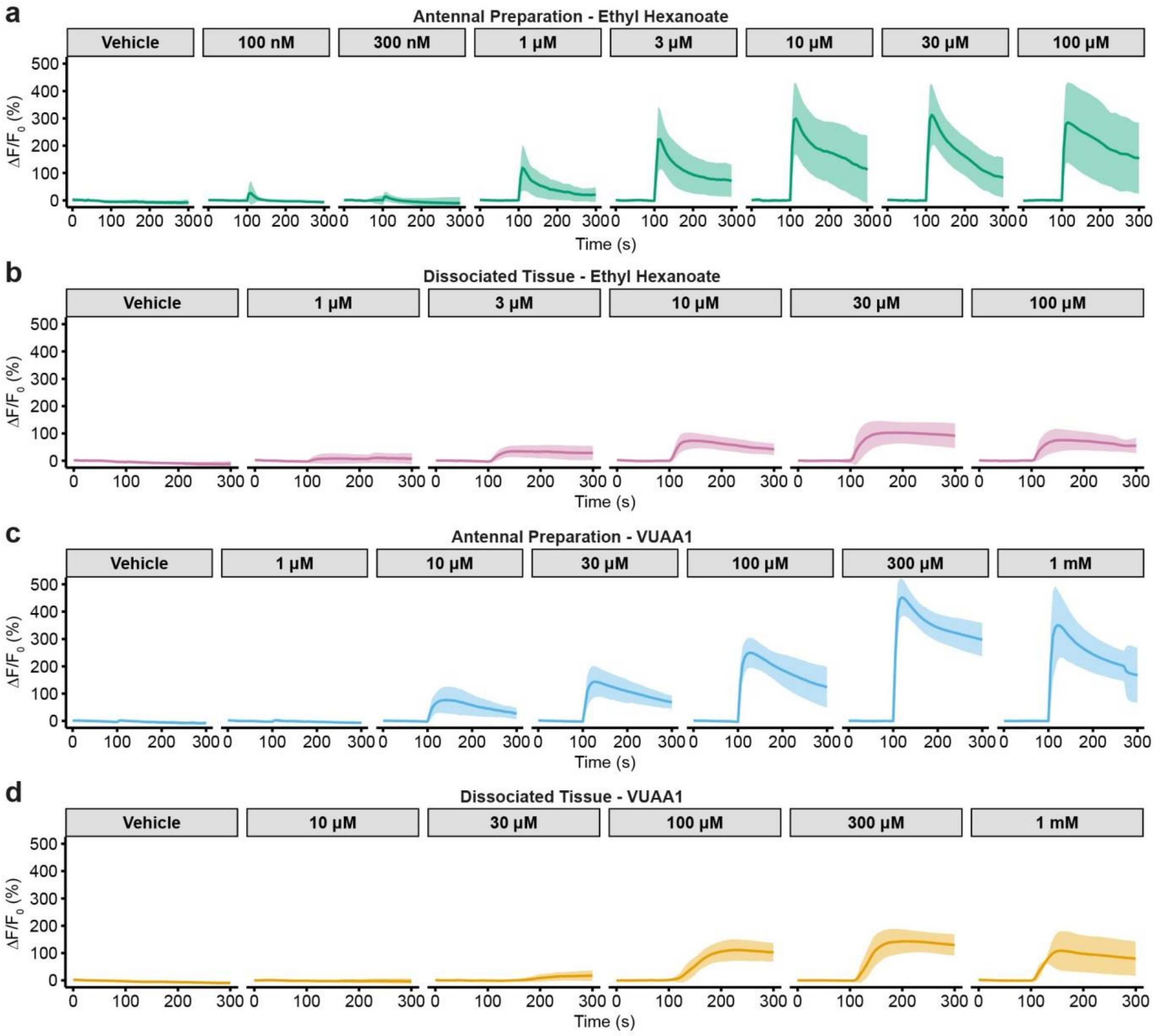

Supplementary Figure 5| Response profile of *D. melanogaster* OSNs from undigested antennal preparations and dissociated antennal tissues stimulated with OR agonists.

Ethyl hexanoate and VUAA1 elicited concentration-dependent responses in both the antennal preparation and dissociated tissue, although response profiles including the maximum response intensity and response decay time are different between these two conditions. All graphs show mean ± SD; (a) $5 \leq n \leq 11$, (b) $8 \leq n \leq 10$, (c) $5 \leq n \leq 11$, (d) $4 \leq n \leq 11$ for each concentration.

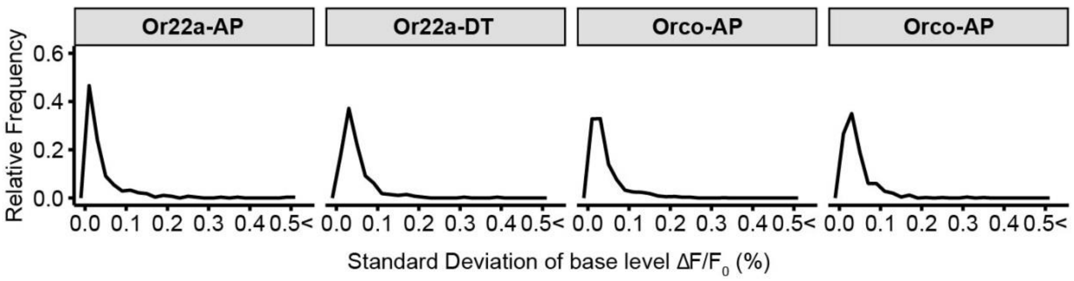

Supplementary Figure 6 | Distribution of standard deviation $\Delta F/F_0$ basal values for analyzed OSN ROIs.

Relative frequency distribution of standard deviation of $\Delta F/F_0$ basal levels before OR agonist stimulation. Antennal preparation from Or22a OSNs (Or22a-AP): 270 ROIs from 71 independent experiments; dissociate tissue from Or22a OSNs (Or22a-DT): 272 ROIs from 60 independent experiments; antennal preparation from all Orco-expressing OSNs (Orco-AP): 924 ROIs from 60 independent experiments; dissociated tissue from Orco OSNs (Orco-DT): 499 ROIs from 49 independent experiments.

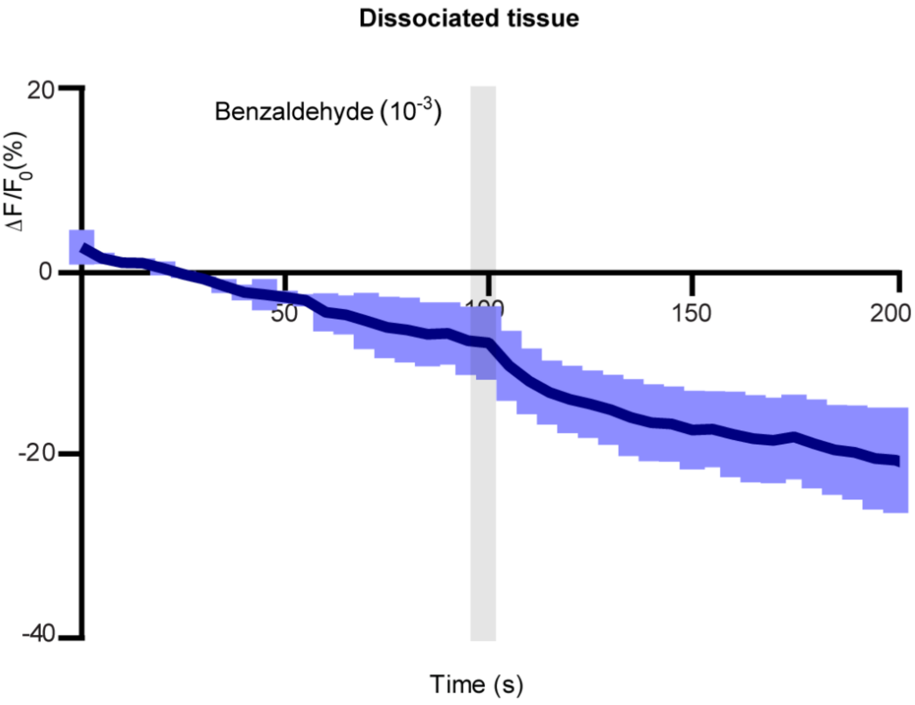

Supplementary Figure 7 | Response profile of OSNs from dissociated antennal tissues of Or22a-GCaMP6f *D. melanogaster* fly.

Time course of Ca^{2+} fluorescence response obtained in Or22a GCaMP6f OSNs upon stimulation with benzaldehyde at 10^{-3} dilution (grey bar). This graph show mean ± SD; n=6

Treatments		t statistics (Welch's t-test)	95% Conficence Interval	df	Uncorrected p value
Alginate vs. Na_2SiO_3	0%	1.732	-1.484, 3.484	2.000	0.2254
	0.5%	12.075	6.601, 11.399	2.941	0.001343
	1.0%	22.000	17.697, 26.303	2.000	0.00206
	1.5%	29.445	30.452, 37.548	3.200	5.264e-05
	2.0%	67.529	47.934, 52.732	2.941	8.694e-06
Agarose vs. Na_2SiO_3	0%	-1.000	-3.535, 2.202	2.000	0.4226
	0.5%	4.810	1.261, 10.739	2.941	0.001343
	1.0%	6.003	5.765, 17.569	3.275	0.00718
	1.5%	8.699	12.357, 26.976	2.919	0.003553
	2.0%	24.981	30.056, 38.610	3.124	0.0001057

Supplementary Table 1 | Statistical analysis of Supplementary Figure 2b.

Treatment	t statistics (Welch's t-test)	95% Conficence Interval	df	Uncorrected p value
0 vs 10 min	1.265	-2.503, 5.170	2.439	0.3133
0 vs. 30 min	0.277	-3.208, 3.874	3.484	0.7972
0 vs. 60 min	0.000	-4.303, 4.303	2.000	1.000

Supplementary Table 2 | Statistical analysis of Supplementary Figure 2c.

Treatments		W statistics (Wilcoxon test)	95% Conficence Interval	Difference	Uncorrected p value
$[Ca^{2+}]_i$ base level	0 vs 0.5%	49	1.130, 11.320	7.078	0.01399
	0 vs. 1.0%	65	0.735, 10.985	5.703	0.02665
	0 vs. 1.5%	42	-5.000, 5.890	1.855	0.3282
	0 vs. 2.0%	26	-6.030, 2.040	-2.348	0.3704
S. D. of $[Ca^{2+}]_i$	0 vs 0.5%	44	-0.030, 0.330	0.104	0.07211
	0 vs. 1.0%	56	-0.056, 0.304	0.140	0.1728
	0 vs. 1.5%	51	0.026, 0.447	0.278	0.04988
	0 vs. 2.0%	28	-0.214, 0.182	-0.054	0.4807

Supplementary Table 3 | Statistical analysis of Supplementary Figure 2d, e.

Time after stimulation (s)	MO (mean ± SEM)	EH (mean ± SEM)	t	df	p value
**10**	-0.4224 ± 0.3465	2.982 ± 1.064	3.281	4	0.0305
**12.5**	-0.6814 ± 0.5194	3.404 ± 0.901	5.11	4	0.0069
**15**	-0.9627 ± 0.7789	3.712 ± 1.048	4.661	4	0.0096
**20**	-1.285 ± 1.197	3.756 ± 1.244	3.399	4	0.0273
**25**	-1.421 ± 1.582	3.429 ± 1.605	2.131	4	0.1

Supplementary Table 4 | Statistical analysis of Figure 2i

Preparation	Parameter	Estimate	St. error	t value	p (>|t|)
Or22a-AP	Upper limit	300.98	15.58	19.32	< 2.2e-16
	Slope	-3.86	0.93	-4.17	5.56e-05
	EC_{50} (log)	-5.84	0.08	-73.31	< 2.2e-16
Or22a-DT	Upper limit	88.07	17.43	5.05	1.50e-6
	Slope	-4.24	3.50	-1.21	0.228
	EC_{50} (log)	-5.41	0.25	-21.71	< 2.2e-16
Orco-AP	Upper limit	399.62	20.81	19.21	< 2.2e-16
	Slope	-2.80	0.43	-6.55	2.32e-09
	EC_{50} (log)	-4.30	0.07	-60.88	< 2.2e-16
Orco-DT	Upper limit	103.21	24.76	4.17	6.39e-05
	Slope	-8.78	26.19	-0.335	0.738
	EC_{50} (log)	-3.97	0.15	-26.82	< 2.2e-16

Supplementary Table 5 | Curve fitting parameters for ethyl hexanoate and VUAA1 dose-response curves.

Curve fitting of concentration-dependent responses in Figure 3 (b, d, f, h) was performed using three-parameter logistic models (lower limit = 0), after logarithmic transformation of concentration values, with the R drc package (see Methods section in the main text)

Treatment	t statistics (Welch's t-test)	95% Conficence Interval	df	p value
Or22a DT vs. AP	-0.1214	-0.0120, 0.0106	118.80	0.9036
Orco DT vs. AP	-0.0231	-0.0080, 0.0079	105.61	0.9816

Supplementary Table 6| Statistical analysis of Figure 3i.

Treatment	Estimate	Standard error	t value	p value
Or22a DT vs. AP	1.0787	0.0518	1.5189	0.1313
Orco DT vs. AP	1.0817	0.0441	1.8529	0.06676

Supplementary Table 7| Statistical analysis of Figure 3j.

Supplementary Video 1 | Functional calcium imaging on Or22a olfactory sensory neurons from *D. melanogaster* dissociated antennal tissue.

Calcium imaging from the same preparation shown in Figure 3a. The left panel shows the raw fluorescence intensity expressed in arbitrary units (counts), the right panel shows the variation of fluorescence intensity respect to base level expressed in percentage (% $\Delta F/F_0$). The stimulus consisted of 100 µM ethyl hexanoate.
